# Multi-feature integrated machine learning prediction model for early nephropathy in elderly living with type 2 diabetes mellitus

**DOI:** 10.3389/fendo.2025.1660903

**Published:** 2026-01-21

**Authors:** Tingting Fang, Yuanyuan Yang, Feng Zhuo, Xinran Xie, Jialun Song, Linghua Kong

**Affiliations:** 1 School of Nursing and Rehabilitation, Shandong University, Jinan, China; 2 Marine Engineering College of Dalian Maritime University, Dalian, China; 3 Department of Gynecology, Reproductive Hospital affiliated to Shandong University, Jinan, China

**Keywords:** machine learning, prediction model, early nephropathy, type 2 diabetes mellitus, elderly

## Abstract

**Aims:**

To develop and validate a multi-feature machine learning (ML) model for early diabetic nephropathy (DN) prediction in elderly living with type 2 diabetes mellitus (T2DM), incorporating clinical indicators, symptoms of traditional Chinese medicine (TCM), and ultrasonic imaging features.

**Methods:**

The valid data (including clinical indicators, TCM symptoms, and ultrasonic imaging features) of 786 patients was retained, and the data were divided into training and validation set. Three models were constructed to examine the model’s performance. The optimal indicators were selected for seven ML. Performance was assessed using accuracy, precision, recall, F1 score, and the area under the receiver operating characteristic curve (AUC). The subgroup analysis was conducted based on age.

**Results:**

The multi-feature model, combining clinical data, TCM symptoms, and ultrasound imaging, demonstrated the best performance. Among the ML algorithms, RF exhibited superior performance with an AUC of 0.894, sensitivity of 0.667, specificity of 0.877, precision of 0.769, recall of 0.667, and F1 score of 0.714 in the validation set. Subgroup analysis revealed that the AUC values exceed 0.7 in each group.

**Conclusion:**

This study is the first to incorporate TCM symptoms and ultrasound imaging features into a predictive model for early DN in elderly living with T2DM. The models demonstrated strong predictive performance across different age groups. These findings underscore the potential of early screening, prevention, and intervention in improving outcomes for elderly living with T2DM, offering a novel approach to managing diabetic nephropathy.

## 1 Introduction

The prevalence of diabetes has grown significantly with aging populations, posing major global health challenges. Older adults are disproportionately affected, and this trend is particularly evident in China, which, according to the 2019 International Diabetes Federation (IDF), is home to approximately 35.5 million individuals aged 65 and elderly living with diabetes—accounting for nearly a quarter of the global elderly diabetic population (1, 2). Type 2 diabetes mellitus (T2DM) constitutes the majority of these cases, representing 90–95% of all diabetes diagnoses (3). Among elderly living with T2DM, the risk of diabetic nephropathy (DN), a severe complication, is alarmingly high, affecting 21.8% of this population in China (4). Furthermore, DN prevalence in individuals over 60 years of age is estimated to be between 20% and 40%, making it the leading cause of end-stage renal disease (ESRD) (4).

The burden of DN continues to rise, with projections indicating over 24.3 million T2DM-related DN cases in China alone (5). Early DN is characterized by subtle and often atypical symptoms, leading to delayed detection. Without timely intervention, DN can progress to significant proteinuria and ESRD, the latter occurring at a rate 14 times higher than other kidney diseases once DN advances to its later stages (4). As such, the early stage of DN represents a critical window for intervention, where timely screening and preventive strategies have the potential to alter the disease trajectory (4, 6, 7).

Although several predictive models, such as the RECODe model (8), UKPDS outcomes model 2 (9), and the Renal DCS Risk Score (10), have been developed, these are predominantly designed for patients with advanced renal disease. Models specifically addressing early DN are rare and often rely on diagnosis markers, such as estimated glomerular filtration rate (eGFR) and urinary albumin-to-creatinine ratio (UACR) (7, 11–16).

Emerging evidence suggests that incorporating TCM symptoms into prediction models offers unique advantages. Unlike conventional biochemical indicators, TCM symptomology provides a holistic view of the patient’s health status, offering insights into subtle pathophysiological changes that might precede biochemical abnormalities (17, 18). Similarly, ultrasonography is increasingly recognized for its potential in stratifying the risk of early DN by assessing both renal and systemic vascular health. Beyond emerging renal techniques (e.g., elastography for stiffness and Doppler for intrarenal hemodynamics), carotid ultrasound findings—such as increased carotid intima-media thickness (cIMT) and the presence of carotid artery plaque—have been shown to be independent risk factors for the development and progression of DN in T2DM patients. This underscores the value of ultrasound in providing a non-invasive, integrative evaluation of the cardiorenal system (19–24). The integration of these multidimensional data sources presents an opportunity to enhance the predictive accuracy and clinical utility of DN models.

This study aims to fill these critical gaps by developing a comprehensive, multi-feature machine learning (ML) prediction model for early DN in elderly living with T2DM. By combining clinical indicators, TCM symptoms, and ultrasound imaging features, the study seeks to identify the most predictive variables and the optimal ML algorithm for early DN detection. This innovative approach offers a timely and significant contribution to advancing early screening, diagnosis, and management of DN, addressing a pressing need in the care of aging populations at high risk for kidney disease.

## 2 Methods

This study was approved by the Ethics Committee of The Affiliated Hospital of Hangzhou Normal University (Approval No. 2022KS034) and adhered to the principles outlined in the Declaration of Helsinki. The study followed the Transparent Reporting of a Multivariable Prediction Model for Individual Prognosis or Diagnosis (TRIPOD) guidelines. Informed consent was submitted by all subjects when they were enrolled.

### 2.1 Study design and patient selection

**Figure 1:**
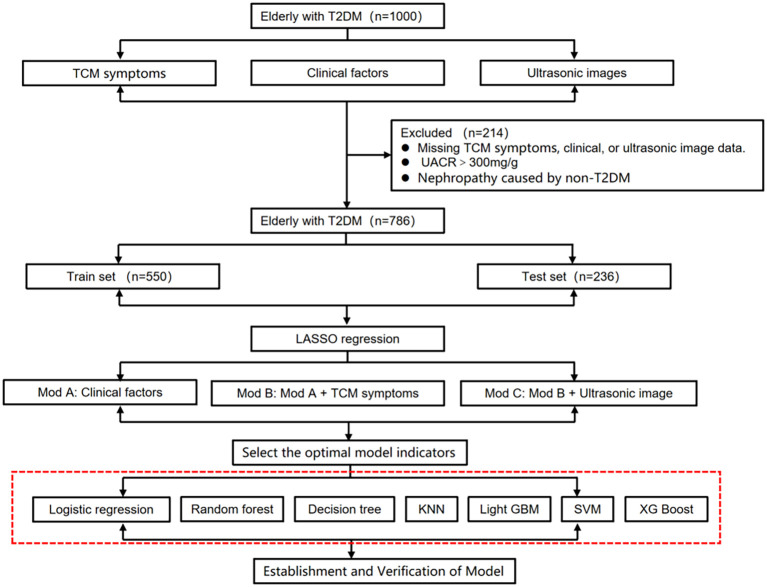
Research flow chart.

Elderly living with T2DM were recruited from The Affiliated Hospital of Hangzhou Normal University between May 2021 and October 2022. The inclusion criteria were (1): age ≥ 60 years, (2) diagnosis of T2DM, and (3) urinary albumin-to-creatinine ratio (UACR) < 300 mg/g. Patients were excluded if they: (1) were under 60 years of age, (2) had non-T2DM diagnoses, (3) had UACR ≥ 300 mg/g, uremia, or renal failure, (4) had severe infections, systemic immune diseases, organ failure, or malignancies, or (5) lacked essential clinical information. After applying these criteria, 786 patients were included in the study (**Figure 1**).

### 2.2 Diagnostic criteria

T2DM and early DN were diagnosed according to the 1999 World Health Organization criteria and Kidney Disease: Improving Global Outcomes (KDIGO) guidelines (25, 26). Early DN was defined as a UACR between 30–300 mg/g, confirmed by at least two of three tests conducted over 3–6 months.

### 2.3 Data collection

Patient data were obtained from the hospital’s electronic medical records and a questionnaire survey. The data were categorized into three domains:

#### 2.3.1 Clinical information

Variables included gender, age, smoking, drinking, comorbidities (hypertension, coronary heart disease, cerebral infarction, hyperlipidemia), family history, diabetic retinopathy, diabetes duration, systolic and diastolic blood pressure, body mass index (BMI), and laboratory parameters such as bilirubin (total, direct, and indirect), calcium, blood urea nitrogen, serum creatinine, uric acid, retinol-binding protein, cholesterol (total, triglycerides, HDL-c, LDL-c), C-reactive protein (CRP), red cell distribution width, platelet distribution width, platelet-to-lymphocyte ratio, high-sensitivity CRP (hs_CRP), glycated hemoglobin (HbA1c), and UACR.

#### 2.3.2 Traditional Chinese medicine symptoms

A TCM symptom questionnaire was designed based on diagnostic and syndrome differentiation criteria (27–29).Ten variables commonly associated with DN were assessed, including fatigue, night sweats, cold extremities, face and feet edema, lumbar and knee weakness, tinnitus and deafness, insomnia and dreaminess, blurred vision, nocturnal polyuria, limb numbness. Responses were scored as “Yes” (1) or “No” (0) (30, 31).

#### 2.3.3 Ultrasound imaging indicators

Variables included left ventricular diastolic dysfunction decreased, valvular regurgitation, renal cysts, nephrolithiasis, renal parenchymal calcification, carotid intima thickening, and carotid artery plaque.

### 2.4 Data cleaning

Data were subjected to an extensive cleaning process to ensure quality and completeness. Records with missing values exceeding 15% were excluded (32, 33), while missing values under 15% were addressed using simple interpolation. We used SPSS 22.0 for simple interpolation (< 15% missing): imputation methods (1): continuous: series mean imputation; (2) categorical: mode imputation. Duplicate and extreme outliers were removed.

### 2.5 Statistical analysis

Statistical analyses were conducted using SPSS 22.0 and R software (v4.2.0). Continuous variables with normal distributions were compared using t-tests or ANOVA and reported as mean ± standard deviation (SD). Non-normally distributed data were analyzed with non-parametric tests (Wilcoxon rank-sum or Kruskal-Wallis H test) and reported as medians with interquartile ranges (P25, P75). Categorical variables were compared using chi-square or Fisher’s exact tests and expressed as counts and percentages. A two-tailed p-value < 0.05 was considered statistically significant.

### 2.6 Feature selection and model evaluation

Least absolute shrinkage and selection operator (LASSO) regression was performed to identify key predictors, followed by multivariate logistic regression (34). A stepwise backward elimination method was used to finalize the model variables. Model discrimination was evaluated using receiver operating characteristic (ROC) curves, with the area under the curve (AUC) calculated using the “pROC” package in R. AUC comparisons between models were conducted with the DeLong test. Calibration and clinical utility were assessed using calibration curves and decision curve analysis (DCA), with the “rms” and “nricens” packages, respectively. Nomograms were constructed for visualization.

### 2.7 Machine learning algorithms

The final model variables were used to develop predictive models using seven machine learning algorithms: logistic regression (LR), decision tree (DT), random forest (RF), k-nearest neighbors (KNN), light gradient boosting machine (Light GBM), support vector machine (SVM), and XGBoost (XGB). The performance of each algorithm was evaluated using accuracy, precision, recall, F1 score, and AUC.

## 3 Results

### 3.1 Baseline characteristics of participants

A total of 786 participants were included in this study, with 550 allocated to the training set and 236 to the validation set in a 7:3 ratio. Baseline comparisons revealed significant differences in the variables “cold extremities”, diabetes duration, total bilirubin (TB), and indirect bilirubin (IB) between the training and validation sets (P < 0.05). No significant differences were observed for other variables (P > 0.05), confirming the comparability between the training and validation sets (**Table 1**).

**Table 1:** Comparison of baseline data between training and validation sets.

Variable	ALL (N = 786)	Training set (N = 550)	Validation set (N = 236)	P
Age	68.00 [64.00,76.00]	69.00 [64.00,76.00]	68.00 [63.00,77.00]	0.745
Gender				0.554
male	412 (52.42%)	284 (51.64%)	128 (54.24%)	
malefemale	374 (47.58%)	266 (48.36%)	108 (45.76%)	
Smoking	178 (22.65%)	123 (22.36%)	55 (23.31%)	0.845
Drinking	150 (19.08%)	104 (18.91%)	46 (19.49%)	0.927
Fatigue	506 (64.38%)	355 (64.55%)	151 (63.98%)	0.944
Night sweats	105 (13.36%)	76 (13.82%)	29 (12.29%)	0.643
Cold extremities	280 (35.62%)	179 (32.55%)	101 (42.80%)	0.008
Face and feet edema	51 (6.49%)	33 (6.00%)	18 (7.63%)	0.490
Lumbar and knee weakness	351 (44.66%)	234 (42.55%)	117 (49.58%)	0.082
Tinnitus and deafness	437 (55.60%)	297 (54.00%)	140 (59.32%)	0.194
Insomnia and dreaminess	346 (44.02%)	238 (43.27%)	108 (45.76%)	0.571
Blurred vision	505 (64.25%)	353 (64.18%)	152 (64.41%)	1.000
Nocturnal polyuria	358 (45.55%)	241 (43.82%)	117 (49.58%)	0.159
Limb numbness	346 (44.02%)	238 (43.27%)	108 (45.76%)	0.571
Hypertension	568 (72.26%)	398 (72.36%)	170 (72.03%)	0.994
CHD	305 (38.80%)	210 (38.18%)	95 (40.25%)	0.641
Cerebral infarction	208 (26.46%)	145 (26.36%)	63 (26.69%)	0.993
Hyperlipidemia	306 (38.93%)	205 (37.27%)	101 (42.80%)	0.169
Family history	327 (41.60%)	223 (40.55%)	104 (44.07%)	0.401
DR	303 (38.55%)	216 (39.27%)	87 (36.86%)	0.578
Diabetes duration	10.00 [6.00,20.00]	10.00 [7.00,20.00]	10.00 [5.00,16.25]	0.032
SBP	138.00 [126.00,153.00]	138.00 [126.00,154.00]	137.00 [127.00,151.00]	0.616
DBP	76.00 [68.00,83.00]	75.00 [68.00,83.00]	77.00 [70.00,84.00]	0.076
BMI	23.95 [22.14,25.77]	23.95 [22.04,25.70]	23.95 [22.57,25.90]	0.315
TB	12.50 [9.50,16.30]	12.10 [9.43,15.90]	13.10 [10.10,17.90]	0.015
DB	2.60 [1.90,3.50]	2.60 [1.90,3.40]	2.60 [1.90,3.80]	0.513
IB	9.80 [7.40,13.07]	9.50 [7.30,12.30]	10.60 [7.70,14.03]	0.007
Ca	2.27 [2.18,2.34]	2.27 [2.18,2.34]	2.28 [2.19,2.35]	0.444
BUN	5.94 [4.76,7.50]	6.08 [4.88,7.59]	5.54 [4.60,7.41]	0.097
SCr	71.55 [56.60,90.68]	71.75 [57.65,90.45]	69.00 [55.58,91.12]	0.416
UA	322.79 [260.00,392.00]	323.50 [266.00,390.00]	321.00 [247.00,393.00]	0.494
RBP	43.63 [35.50,51.40]	43.63 [35.73,51.85]	43.00 [33.98,50.62]	0.206
TC	4.20 [3.41,4.93]	4.18 [3.40,4.93]	4.24 [3.45,4.92]	0.636
TG	1.34 [0.96,1.87]	1.30 [0.95,1.85]	1.44 [1.01,1.91]	0.154
HDL-c	1.11 [0.92,1.30]	1.12 [0.92,1.30]	1.09 [0.92,1.27]	0.761
LDL-c	2.44 [1.88,2.96]	2.42 [1.88,2.96]	2.46 [1.87,2.96]	0.830
CRP	2.30 [1.20,7.10]	2.30 [1.20,7.27]	2.20 [1.08,6.93]	0.637
RDW	12.80 [12.30,13.20]	12.80 [12.30,13.20]	12.70 [12.20,13.20]	0.053
PLR	131.91 [94.65,160.93]	132.14 [94.68,161.79]	128.16 [94.50,159.57]	0.907
hs_CRP	4.00 [1.29,9.32]	4.00 [1.35,9.32]	4.00 [1.27,8.79]	0.287
HbA1c	8.78 [7.30,10.40]	8.78 [7.40,10.38]	8.80 [7.20,10.60]	0.636
Left ventricular diastolic function decreased	356 (45.29%)	259 (47.09%)	97 (41.10%)	0.142
Valvular regurgitation	350 (44.53%)	256 (46.55%)	94 (39.83%)	0.097
Renal cyst	142 (18.07%)	91 (16.55%)	51 (21.61%)	0.112
Nephrolithiasis	87 (11.07%)	63 (11.45%)	24 (10.17%)	0.687
Renal parenchymal calcification	209 (26.59%)	149 (27.09%)	60 (25.42%)	0.691
Carotid intima thickening	309 (39.31%)	206 (37.45%)	103 (43.64%)	0.121
Carotid artery plaque	453 (57.63%)	316 (57.45%)	137 (58.05%)	0.939

CHD, coronary heart disease; DR, diabetic retinopathy; SBP, systolic blood pressure; DBP, diastolic blood pressure; BMI, body mass index; TB, total bilirubin; DB, direct bilirubin; IB, indirect bilirubin; Ca, blood calcium; BUN, blood urea nitrogen; SCr, serum creatinine; UA, uric acid; RBP, retinol-binding protein; TC, total cholesterol; TG, triglycerides; HDL-c, high-density lipoprotein cholesterol; LDL-c, low-density lipoprotein cholesterol; CRP, C-reactive protein; RDW, red cell distribution width; PDW, platelet distribution width; PLR, platelet to lymphocyte ratio; hs_CRP, high sensitivity C-reactive protein; HbA1c, glycated hemoglobin.

### 3.2 Variable selection using LASSO and logistic regression

**Figure 2:**
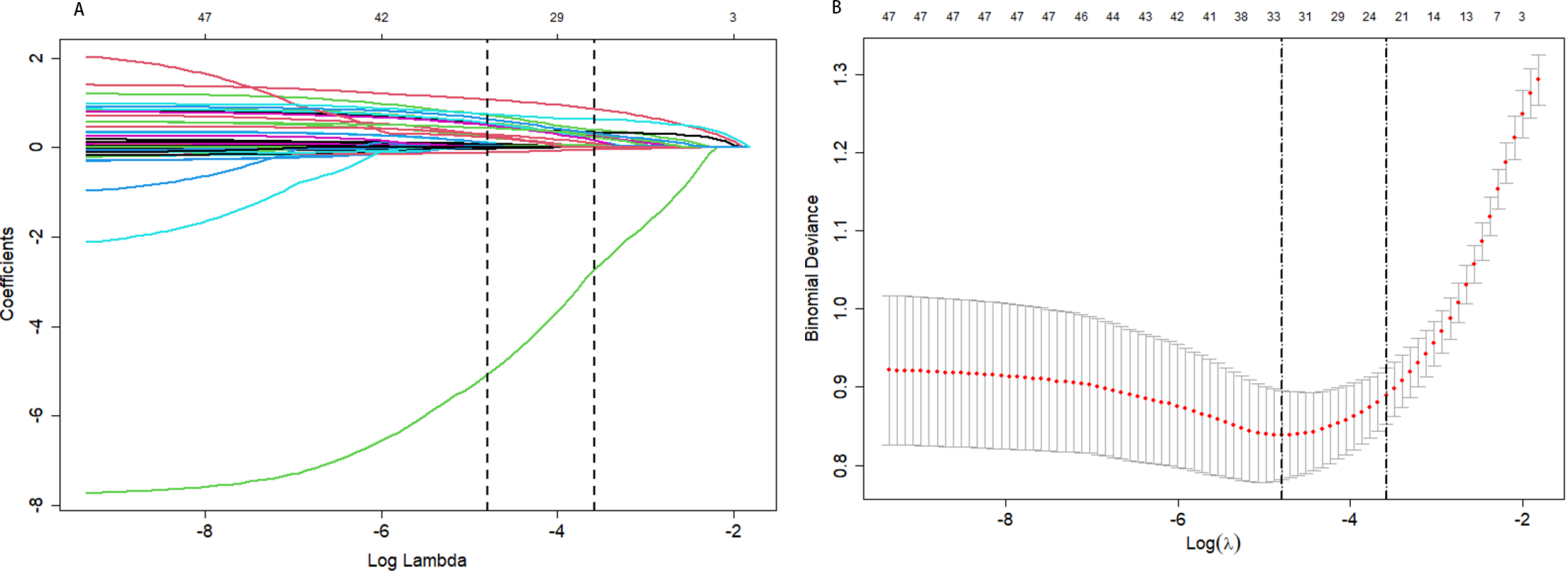
Best match factor screening by lasso regression. **(A)** The Lasso regression path diagram; **(B)** The plot of the best matching factors screened by the ten-fold cross validation method, and the best matching factors were selected using lambda.1se as the criterion.

LASSO regression was applied to identify relevant predictors, using early DN as the dependent variable and 48 candidate variables as independent variables. The optimal model was determined through ten-fold cross-validation (**Figure 2**). LASSO analysis identified 21 significant factors, including cold extremities, lumbar and knee weakness, blurred vision, nocturnal polyuria, limb numbness, hypertension, cerebral infarction, hyperlipidemia, diabetic retinopathy (DR), diabetes duration, systolic blood pressure (SBP), indirect bilirubin (IB), blood calcium (Ca), blood urea nitrogen (BUN), uric acid (UA), retinol-binding protein (RBP), triglycerides (TG), C-reactive protein (CRP), renal parenchymal calcification, carotid intima thickening, and carotid artery plaque.

Through backward stepwise logistic regression, 15 independent risk factors were identified: lumbar and knee weakness, blurred vision, nocturnal polyuria, hypertension, cerebral infarction, hyperlipidemia, DR, SBP, IB, Ca, UA, RBP, CRP, carotid intima thickening, and carotid artery plaque (**Supplementary Figure S1**).

### 3.3 Comparison of predictive models

Three prediction models were developed:

- Mod A: Clinical indicators only (hypertension, cerebral infarction, hyperlipidemia, DR, SBP, IB, Ca, UA, RBP, CRP).
- Mod B: Clinical indicators and TCM symptoms (Mod A variables + lumbar and knee weakness, blurred vision, nocturnal polyuria).
- Mod C: Clinical indicators, TCM symptoms, and ultrasound imaging (Mod B variables + carotid intima thickening, carotid artery plaque).

**Figure 3:**
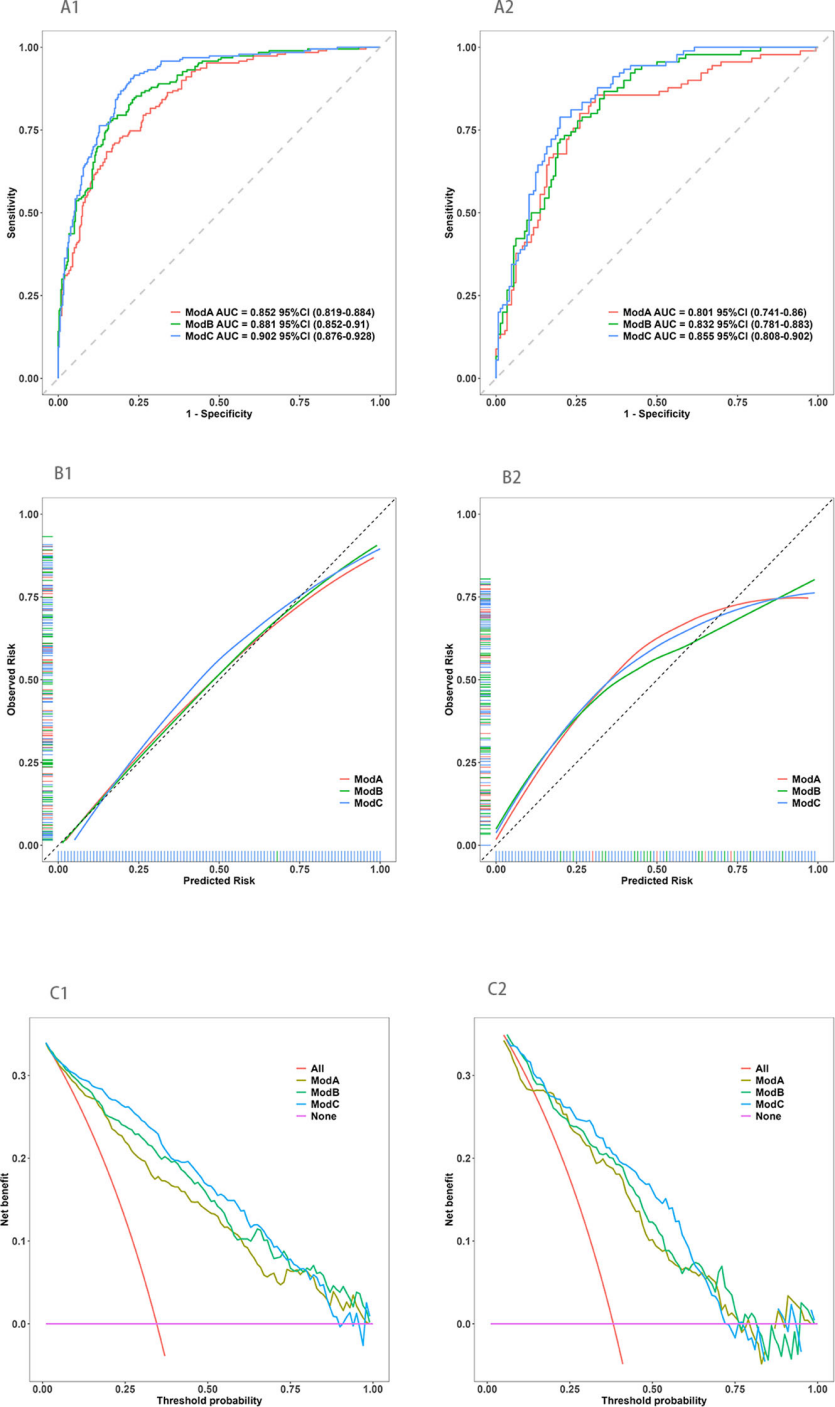
The ROC curve, calibration curve, and the decision curve analysis of three models in the training and validation sets. **(A1, A2)** The ROC curve in the training and validation sets, respectively; **(B1, B2)** The calibration curve in the training and validation sets, respectively; **(C1, C2)** The decision curve analysis in the training and validation sets, respectively.

**Figure 4:**
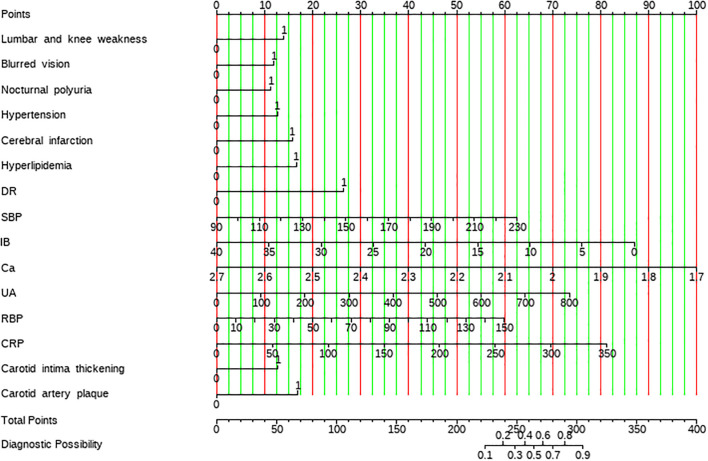
Nomogram of the prediction model for early nephropathy in elderly living with T2DM.

Model performance was evaluated using the area under the receiver operating characteristic curve (AUC). In the training set, AUCs were 0.852 (95% CI: 0.819–0.884) for Mod A, 0.881 (95% CI: 0.852–0.910) for Mod B, and 0.902 (95% CI: 0.876–0.928) for Mod C. In the validation set, AUCs were 0.801 (95% CI: 0.741–0.860), 0.832 (95% CI: 0.781–0.883), and 0.855 (95% CI: 0.808–0.902) for Mod A, Mod B, and Mod C, respectively. Calibration and decision curve analyses further confirm that Mod C exhibited the best predictive performance (**Figure 3**). Based on these findings, Mod C was selected to construct a nomogram for clinical application (**Figure 4**).

### 3.4 Performance of machine learning models

**Figure 5:**
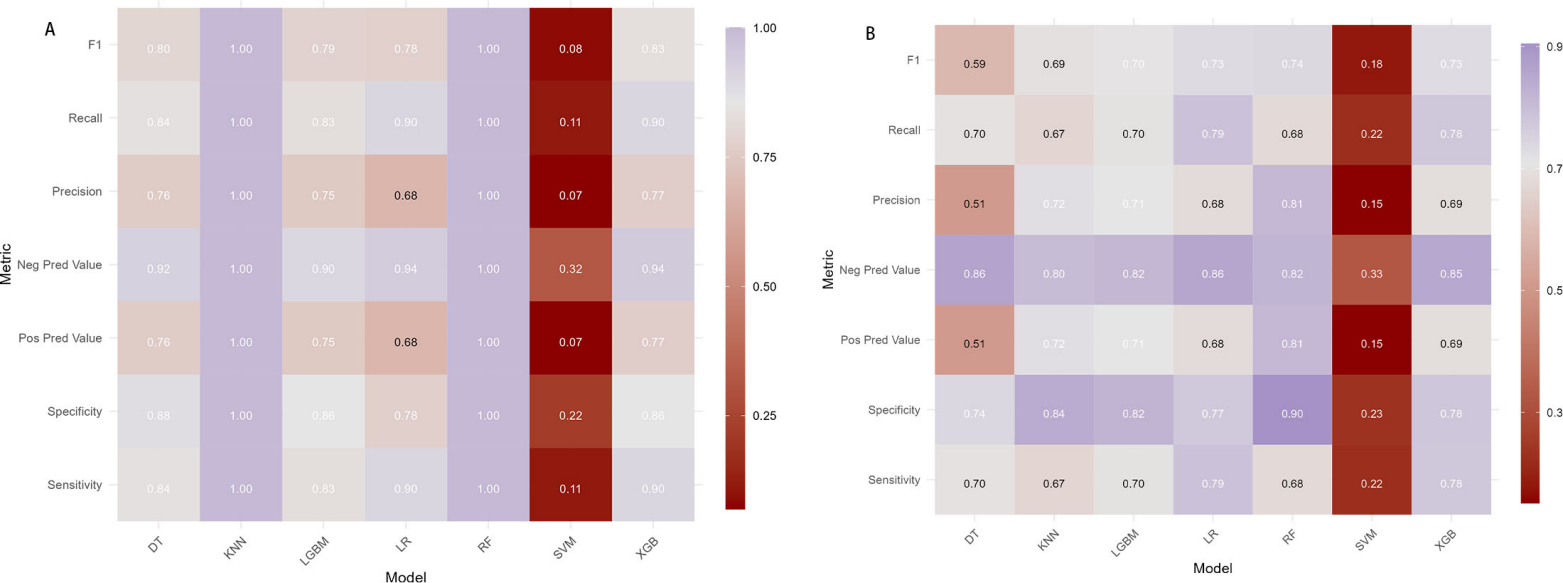
Performance of seven machine learning models between training and validation sets. **(A, B)** Training and validation sets, respectively.

Using the multi-feature data from Mod C, seven machine learning algorithms were evaluated: logistic regression (LR), decision tree (DT), random forest (RF), k-nearest neighbors (KNN), light gradient boosting machine (Light GBM), support vector machine (SVM), and XGBoost (XGB). Among these, RF demonstrated the highest performance. In the training set, RF achieved an AUC, sensitivity, specificity, precision, recall, and F1 score of 1.000. In the validation set, RF yielded an AUC of 0.894, sensitivity of 0.667, specificity of 0.877, precision of 0.769, recall of 0.667, and F1 score of 0.714 (**Supplementary Figure S2**, **Figure 5**, **Table 2**). SHAP analysis identified DR, UA, and carotid artery plaque as the top three predictors influencing early DN in elderly T2DM patients (**Supplementary Figure S3**).

**Table 2:** Performance of seven machine learning models between training and validation sets.

Model	Group	Sensitivity	Specificity	Pos Pred value	Neg Pred value	Precision	Recall	F1	AUC
LR	training set	0.905	0.775	0.680	0.939	0.680	0.905	0.777	0.902
DT	training set	0.838	0.881	0.763	0.922	0.763	0.838	0.799	0.894
RF	training set	1.000	1.000	1.000	1.000	1.000	1.000	1.000	1.000
XGB	training set	0.874	0.922	0.856	0.933	0.856	0.874	0.865	0.955
SVM	training set	0.111	0.219	0.070	0.319	0.070	0.111	0.085	0.902
KNN	training set	1.000	1.000	1.000	1.000	1.000	1.000	1.000	1.000
LGBM	training set	0.826	0.856	0.751	0.903	0.751	0.826	0.787	0.918
LR	validation set	0.789	0.774	0.683	0.856	0.683	0.789	0.732	0.855
DT	validation set	0.697	0.741	0.511	0.863	0.511	0.697	0.590	0.799
RF	validation set	0.667	0.877	0.769	0.810	0.769	0.667	0.714	0.894
XGB	validation set	0.622	0.870	0.747	0.789	0.747	0.622	0.679	0.848
SVM	validation set	0.222	0.233	0.152	0.327	0.152	0.222	0.180	0.852
KNN	validation set	0.667	0.842	0.723	0.804	0.723	0.667	0.694	0.829
LGBM	validation set	0.700	0.822	0.708	0.816	0.708	0.700	0.704	0.837

Pos Pred Value, positive prediction value; Neg Pred Value, negative prediction value; AUC, area under the curve; LM, logistic regression model; DT, decision tree; RF, random forest; XGB, XG Boost; SVM, support vector machine; KNN, k-nearest neighbors, Light GBM, light gradient boosting machine.

### 3.5 Subgroup analysis by age

To account for potential age-related differences in DN, subgroup analyses were performed by stratifying participants into three age groups: 60–69 years, 70–79 years, and ≥ 80 years. In the training set, AUCs were 0.933 (95% CI: 0.902–0.964) for ages 60–69, 0.935 (95% CI: 0.900–0.970) for ages 70–79, and 0.837 (95% CI: 0.756–0.917) for ages ≥ 80. In the validation set, AUCs were 0.879 (95% CI: 0.814–0.944), 0.923 (95% CI: 0.861–0.985), and 0.718 (95% CI: 0.559–0.878), respectively (**Supplementary Figure S4**). Forest plots and age-specific nomograms were generated to visualize risk factors for each age group and to facilitate clinical decision-making (**Supplementary Figures S5**, **S6**).

## 4 Discussion

Numerous established models for predicting diabetic nephropathy (DN) primarily focus on patients with end-stage renal disease or renal failure (8–10). Early DN prediction models, while incorporating diagnostic markers such as estimated glomerular filtration rate (eGFR) and urinary albumin-to-creatinine ratio (UACR), are generally based on demographic, anthropometric, and biochemical indicators (7, 11, 14, 35). In this study, we constructed a novel predictive model integrating clinical data, traditional Chinese medicine (TCM) symptoms, and ultrasound imaging indicators to predict early DN in elderly living with type 2 diabetes mellitus (T2DM).

Among the three models developed, the combined model (Mod C) demonstrated the highest predictive value, with an area under the curve (AUC) of 0.902. This performance highlights the synergistic value of combining clinical data, TCM symptoms, and ultrasound imaging features, surpassing the predictive ability of models based solely on clinical or single-domain data. Our findings align with recent studies emphasizing the importance of integrating TCM symptoms and imaging features in disease prediction (16, 36). Consequently, Mod C was utilized to construct a nomogram, and its performance was further validated using machine learning (ML) algorithms, demonstrating strong discrimination and calibration. Our nomogram-based prediction model can be implemented clinically through the following standardized procedures (1): assess TCM syndromes (using standardized diagnostic scale); (2) obtain ultrasound parameters; (3) input routine metrics (e.g., hypertension, cerebral infarction, hyperlipidemia, DR, SBP, IB); (4) plot scores on corresponding nomogram axes; (5) sum total points to read predicted probability.

### 4.1 Clinical relevance of predictors

The predictive model identified several key factors related to early DN in older patients. TCM symptoms such as lumbar and knee weakness, blurred vision, and nocturnal polyuria emerged as critical predictors. These symptoms reflect the underlying deficiencies in kidney Yin and Yang, which are central to TCM pathophysiology (37, 38). TCM classifies DN based on syndrome differentiation (e.g., Qi-Yin deficiency), which reflects systemic pathological changes even in early-stage DN when conventional biomarkers (e.g., microalbuminuria) may still be within normal ranges (39). Lumbar and knee weakness, blurred vision, and nocturnal polyuria may serve as early warning signs, aiding in the identification of high-risk patients before structural kidney damage becomes evident. Age-related declines in kidney function further exacerbate these deficiencies, as documented in prior studies (40, 41).

Ultrasound imaging indicators, particularly carotid intima thickening and carotid artery plaque, were also identified as significant predictors. These markers are commonly associated with diabetic macroangiopathy and atherosclerosis, which are closely linked to DN progression (42–48). Although not a gold standard, ultrasound provides valuable structural and hemodynamic insights, such as: carotid intima thickening and carotid artery plaque (suggesting diabetic macroangiopathy and atherosclerosis) (49). These findings, combined with laboratory tests, enhance early DN detection sensitivity. While the relationship between carotid intima-media thickness (IMT) and DN remains controversial, our study reinforces its role as an independent risk factor for early DN. TCM syndrome progression (e.g., from Qi-Yin deficiency to Yang deficiency) correlates with ultrasound-documented structural decline (e.g., carotid intima thickening and carotid artery plaque), offering a holistic view of disease progression. We acknowledge the need for further research on correlations between specific TCM syndromes (e.g., Spleen-Kidney Qi deficiency) and ultrasound parameters (e.g., carotid intima thickening and carotid artery plaque). Prospective studies validating a combined TCM-ultrasound predictive model would strengthen clinical utility.

Clinical factors such as hypertension, cerebral infarction, hyperlipidemia, diabetic retinopathy (DR), systolic blood pressure (SBP), indirect bilirubin (IB), calcium (Ca), uric acid (UA), retinol-binding protein (RBP), and C-reactive protein (CRP) were also significant predictors. Known risk factors, including DR, hypertension, and hyperlipidemia, align with established evidence (7, 11, 50). Notably, UA, an oxidative stress marker, is strongly associated with proteinuria, glomerular filtration rate decline, and DN progression (51–54). RBP, an early diagnostic marker of proximal tubular dysfunction, also emerged as a significant predictor (55). Interestingly, IB, with its antioxidant properties, was identified as a protective factor against DN, consistent with emerging research on its role in mitigating oxidative stress (56). Further studies are warranted to explore the mechanisms underlying these associations.

### 4.2 Subgroup analysis

Given the known epidemiological and physiological differences in DN risk across age groups, subgroup analyses were conducted. Predictive models showed robust performance across all age groups, with AUC values exceeding 0.7 in both training and validation datasets. The performance was particularly strong for patients aged 60–79 years, with slightly lower predictive accuracy for those aged ≥ 80 years, potentially due to smaller sample sizes. These findings emphasize the adaptability and applicability of the model for various age cohorts, providing a practical tool for early DN risk stratification in older populations.

### 4.3 Machine learning model comparison

The integration of clinical, TCM, and ultrasound imaging data was further validated through ML approaches. Among the seven algorithms evaluated, the random forest (RF) model exhibited the best predictive performance, with an AUC of 0.894, sensitivity of 0.667, specificity of 0.877, precision of 0.769, recall of 0.667, and F1 score of 0.714 in the validation set. These results confirm RF’s robustness, aligning with prior research that highlighted its superiority in predicting progression to end-stage renal disease (ESRD) (35, 57, 58). Notably, few existing ML models incorporate TCM and imaging data, underscoring the novelty and clinical relevance of our approach. Risk Equations for Complications Of type 2 Diabetes (RECODe) were derived from the Action to Control Cardiovascular Risk in Diabetes (ACCORD) study (59, 60). Compared with established DN prediction tools like RECODe, our model incorporates TCM syndromes (e.g., lumbar and knee weakness, blurred vision, and nocturnal polyuria) and ultrasound parameters (e.g., carotid intima thickening and carotid artery plaque). This multimodal design targets earlier prediction windows than RECODe. At the same time, the RECODe predicts 3–5 year renal function decline, our model identifies pre-clinical risks (TCM manifestations precede lab abnormalities).

### 4.4 Implications and limitations

This study provides a comprehensive predictive framework for early DN in elderly living with T2DM, incorporating multidimensional data to improve accuracy. The integration of TCM symptoms and ultrasound imaging data represents a novel and clinically valuable approach, facilitating early detection and targeted management. However, studying has several limitations.

#### 4.4.1 Single-center and internal validation design

The participants were recruited from a single hospital with internal validation, introducing potential selection bias. Future studies should include multicenter cohorts and external validation to enhance generalizability.

#### 4.4.2 Subjectivity of TCM assessments

Although standardized questionnaires were used, the inherent subjectivity and lack of uniform standards in TCM symptom evaluation remain a challenge. Future work will involve multicenter collaboration and AI-assisted tools (e.g., CNN) to enhance objectivity.

#### 4.4.3 Sample size constraints

The subgroup of patients aged ≥ 80 years was relatively small (n=146 vs. 421 in 60-69y and 219 in 70-79y groups), which may have influenced the predictive performance in this group. Larger studies are needed to validate these findings.

#### 4.4.4 Ultrasound imaging variability

Differences in equipment and operator expertise were not accounted for, potentially affecting the consistency of imaging data. Standardized imaging protocols should be adopted in future studies.

#### 4.4.5 Non-albuminuric nephropathy was not considered

Studies documented that non-albuminuric DKD (eGFR < 60 mL/min/1.73 m^2^ in the absence of albuminuria) occurs relatively frequently in patients with diabetes and its prevalence is increasing. In this study, the diagnosis of early kidney disease is based on UACR. We did not consider non-albuminuric DKD. In future studies, we will focus on the construction of non-albuminuric DKD prediction model.

## 5 Conclusions

This study successfully developed and validated predictive models for early DN in elderly living with T2DM, incorporating clinical, TCM, and ultrasound imaging data. The RF model demonstrated superior predictive performance, and subgroup analyses confirmed its applicability across different age groups. These findings have significant implications for early DN screening, prevention, and management, particularly in aging populations. Further research is needed to address the study’s limitations and to validate the model in diverse clinical settings.

## Data Availability

The raw data supporting the conclusions of this article will be made available by the authors, without undue reservation.
