# Correction: Multi-feature integrated machine learning prediction model for early nephropathy in elderly living with type 2 diabetes mellitus

**DOI:** 10.3389/fendo.2026.1807912

**Published:** 2026-04-13

**Authors:** Tingting Fang, Yuanyuan Yang, Feng Zhuo, Xinran Xie, Jialun Song, Linghua Kong

**Affiliations:** 1 School of Nursing and Rehabilitation, Shandong University, Jinan, China; 2 Marine Engineering College of Dalian Maritime University, Dalian, China; 3 Department of Gynecology, Reproductive Hospital affiliated to Shandong University, Jinan, China

**Keywords:** machine learning, prediction model, early nephropathy, type 2 diabetes mellitus, elderly

Author “Yuanyuan Yang” was erroneously omitted as equal contributing author. The original version of this article has been updated.

