# Supplementary material for: Multi-feature integrated machine learning prediction model for early nephropathy in elderly living with type 2 diabetes mellitus
Source: Front Endocrinol (Lausanne). 2026 Jan 21;16:1660903. doi: 10.3389/fendo.2025.1660903 (PMC12867848; doi:10.3389/fendo.2025.1660903)
Supplement: Supplementary file 1 [file DataSheet1.docx]

**Supplementary material**

Fig. S1. The forest map of independent influencing factors by binary logistic regression analysis.

Fig. S2. The ROC curve, calibration curve, and the decision curve analysis of machine learning in the training and validation sets, respectively. **A1** and **A2**: The ROC curves in the training and validation sets, respectively; **B1** and **B2**: The calibration curves in the training and validation sets, respectively; **C1** and **C2**: The decision curve analysis in the training and validation sets, respectively.

Fig. S3. Graph showing SHAP analysis features of random forest.

Fig. S4. The ROC curve of binary logistics regression of each age group in the training and validation sets, respectively. **A**: 60-67 years old; **B**: 70-79 years old; **C**:≥ 80 years old.

Fig. S5. The forest maps of independent influencing factors by binary logistic regression analysis in each age group. **A**: 60-67 years old; **B**: 70-79 years old; **C**:≥ 80 years old.

Fig. S6. The nomogram models of each age group. **A**: 60-67 years old; **B**: 70-79 years old; **C**:≥ 80 years old.





Fig. S1. The forest map of independent influencing factors by binary logistic regression analysis.


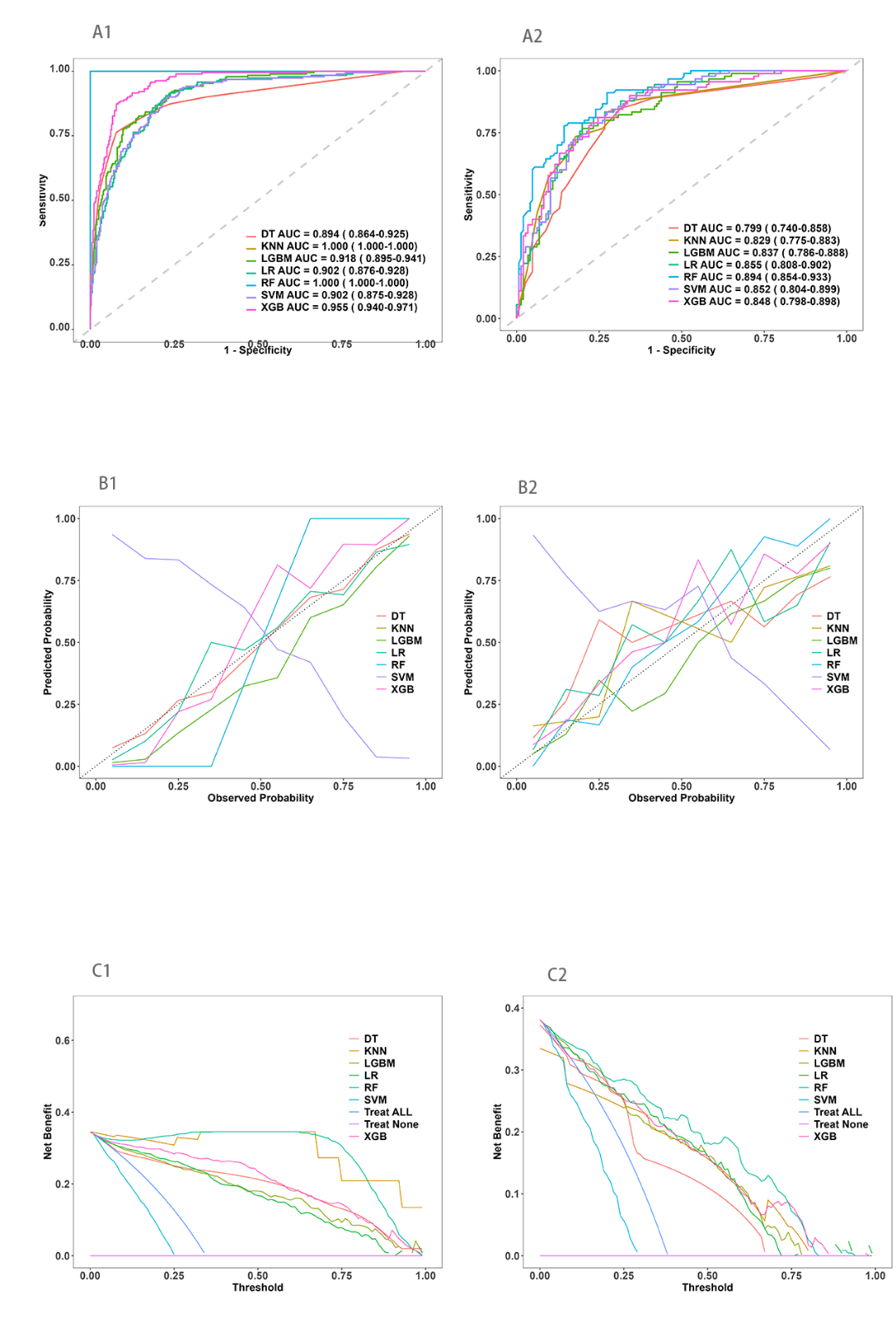


Fig. S2. The ROC curve, calibration curve, and the decision curve analysis of machine learning in the training and validation sets, respectively. **A1** and **A2**: The ROC curves in the training and validation sets, respectively; **B1** and **B2**: The calibration curves in the training and validation sets, respectively; **C1** and **C2**: The decision curve analysis in the training and validation sets, respectively.


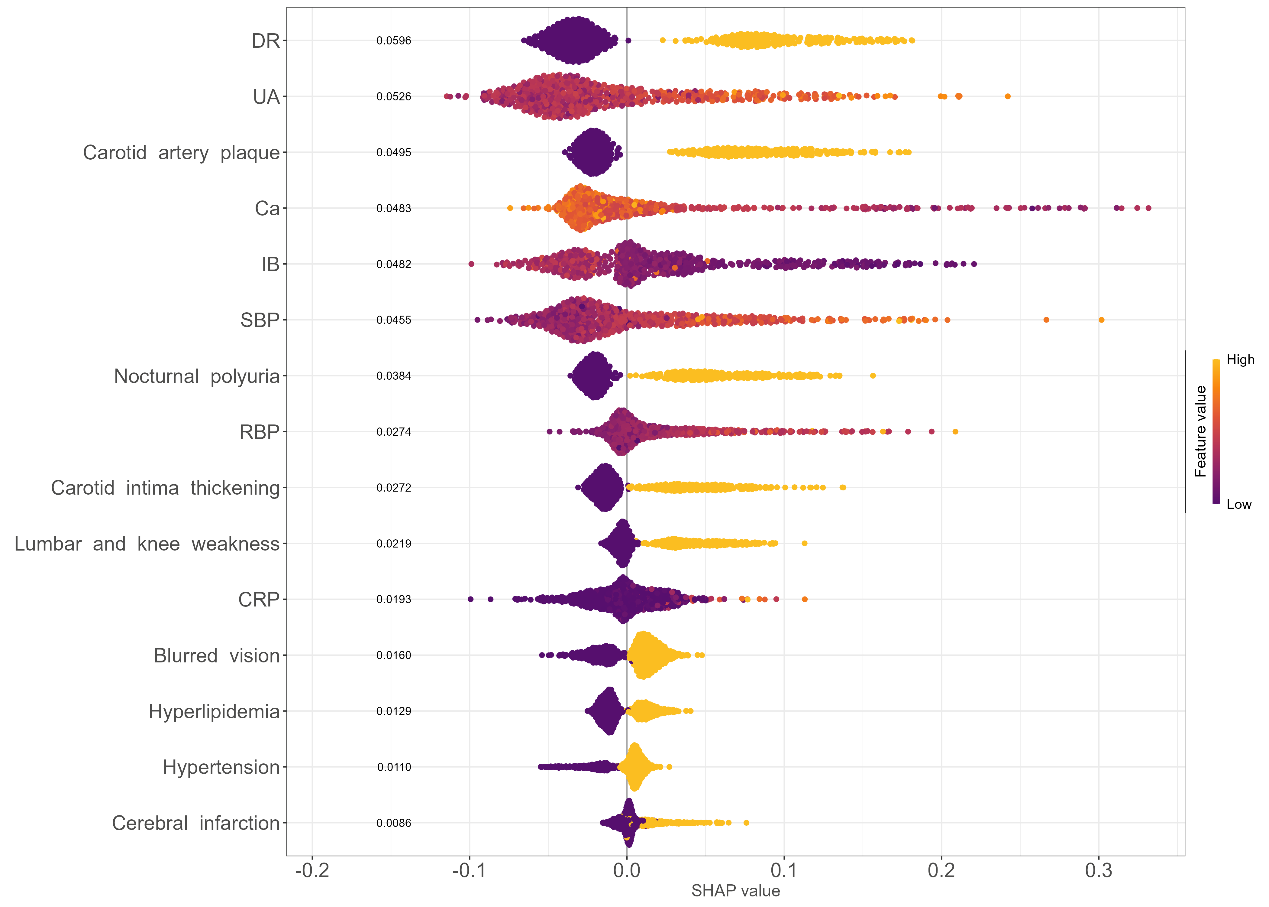


Fig. S3. Graph showing SHAP analysis features of random forest.


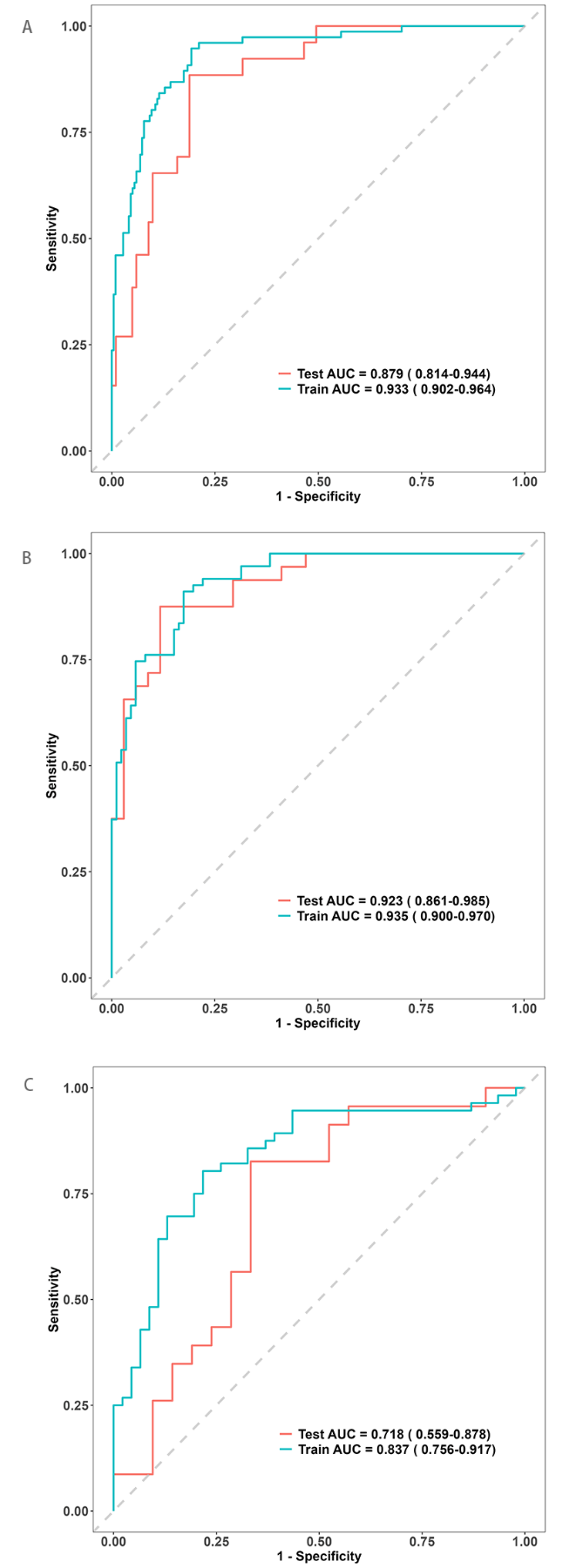


Fig. S4. The ROC curve of binary logistics regression of each age group in the training and validation sets, respectively. **A**: 60-67 years old; **B**: 70-79 years old; **C**:≥ 80 years old.


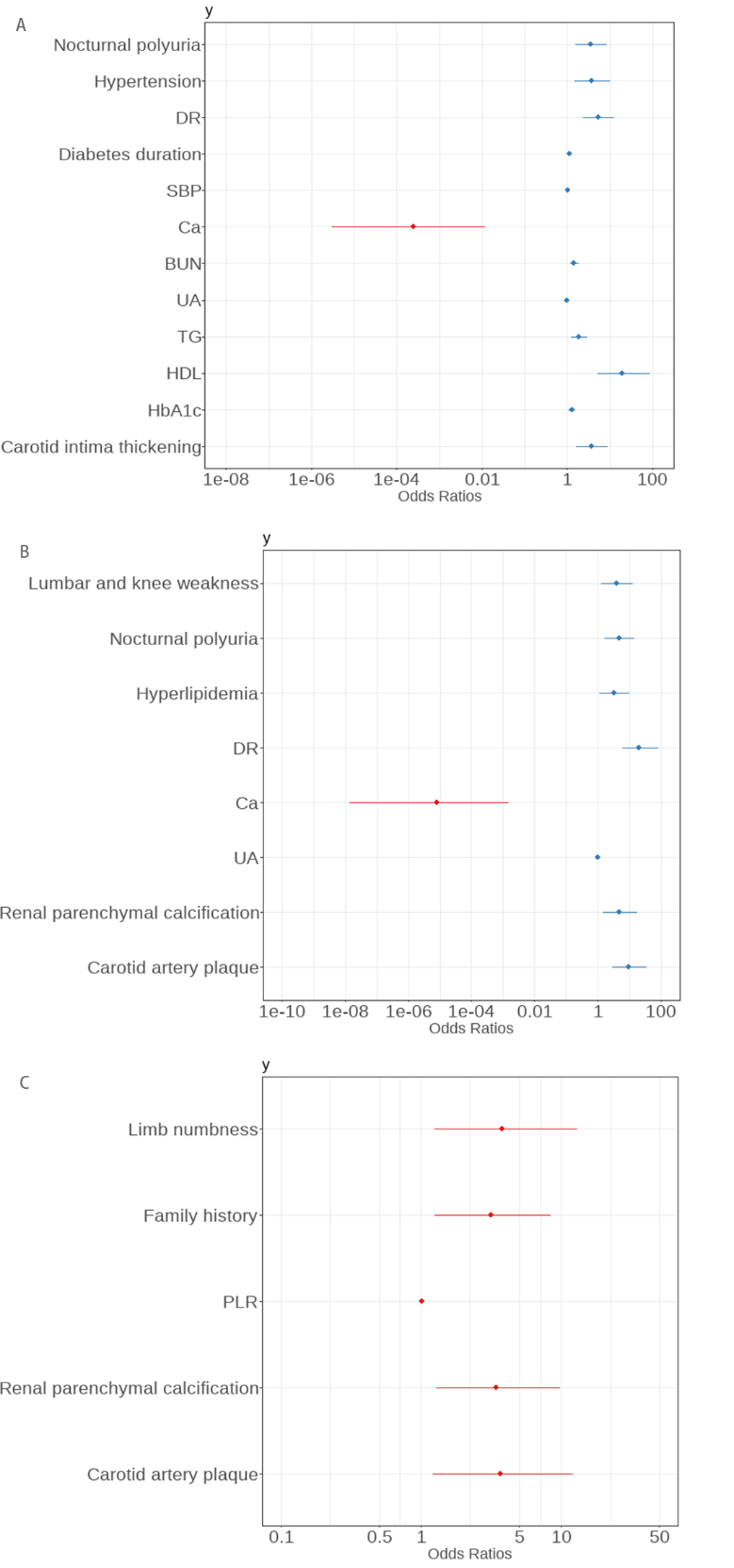


Fig. S5. The forest maps of independent influencing factors by binary logistic regression analysis in each age group. **A**: 60-67 years old; **B**: 70-79 years old; **C**:≥ 80 years old.


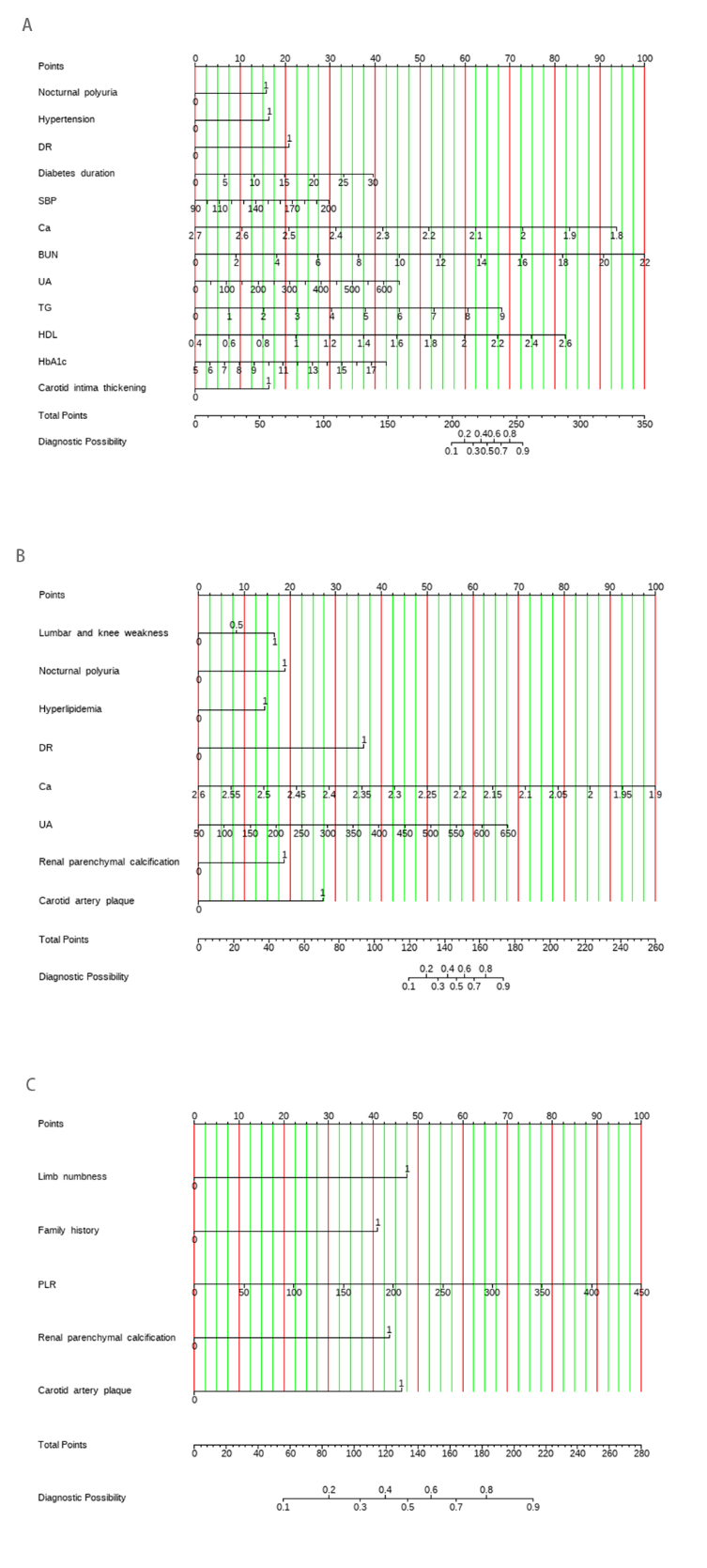


Fig. S6. The nomogram models of each age group. **A**: 60-67 years old; **B**: 70-79 years old; **C**:≥ 80 years old.
